# Paracetamol and the Placebo Effect in Osteoarthritis Trials: A Missing Link?

**DOI:** 10.1155/2011/696791

**Published:** 2011-06-06

**Authors:** Henning Zeidler

**Affiliations:** Hannover Medical School, Carl Neuberg Straße 1, 30625 Hannover, Germany

## Abstract

This paper addresses the role of paracetamol in placebo-controlled osteoarthritis (OA) trials and the potential contribution to the large placebo response in such trials. Paracetamol is used as rescue medication in nearly all OA placebo-controlled trials. Triggered by the discussion about the placebo effect in general and because of the lack of systematic reviews of placebo effect in OA trials, a recent meta-analysis examined the placebo effect and its potential determinants in the treatment of OA, as the main result came out that placebo is very effective in the treatment of OA, especially for pain, stiffness, and self-reported function. However, mostly limited data are available from published OA trials on the starting dose, final dose, dose over time of paracetamol use, and the percentage of patients who used rescue medication during the study. Paracetamol may be an important additional simulated effect of placebo administration mimicking the true placebo effect and thus a missing link contributing partially to the large placebo response in OA trials. Therefore, the positive effect of paracetamol on symptom relief as well as the need for standardized recording of rescue medication should be taken into account when designing, executing, and interpreting placebo-controlled OA studies.

## 1. Introduction

Osteoarthritis (OA) is by far the commonest, chronic, musculoskeletal disorder characterized by joint pain, stiffness, loss of motion, and impaired quality of life afflicting an increasingly older population. There is no curative treatment for this disease despite availability of a large number of therapeutic options, including nonpharmacological, pharmacological, and surgical therapies. Symptomatic treatment to relieve pain and incapacity can be obtained with analgesics such as paracetamol or the more effective nonsteroidal anti-inflammatory drugs (NSAIDs). Current guidelines emphasize paracetamol as the first-line therapy, when pharmacological agents are needed [1–4]. Paracetamol (350 mg up to 4000 mg) is used as rescue medication in nearly all OA trials. It has been speculated whether placebo is effective for OA and which factors may determine the size of such an effect (as discussed by Zhang et al. [5]). This is a common discussion point when a randomised controlled trial (RCT) fails to demonstrate superiority of active treatment over placebo. The present paper addresses the role of paracetamol as rescue medication in OA trials and the potential contribution to the large placebo response in such trials. 

## 2. Paracetamol in Osteoarthritis

Paracetamol (acetaminophen) is a simple analgesic used in OA for decades that has both analgesic and antipyretic actions. It has a narrow therapeutic window but in recommended doses (1 g three to four times daily) is of favourable efficacy and very safe. Studies comparing paracetamol to placebo show that people with OA who take paracetamol have less pain (when resting, moving, sleeping, and overall) and feel better overall than people who take a placebo (as discussed by Towheed et al. [6]). A meta-analysis of randomised controlled trials reported that paracetamol is effective in relieving pain due to OA when used in a fixed dose between 2000 mg and 4000 mg and that paracetamol has a higher response rate than placebo [7]. The effect size (ES) of 0.21 is small but statistically significant, although the efficacy is inferior to that of NSAIDs. A recent Cochrane systemic review concluded that paracetamol is superior to placebo in OA with an improvement from baseline of 5%, an absolute change of 4 points on a 0 to 100 pain scale, and a number needed to treat (NNT) ranging from 4 to 16 [6]. 

One RCT showed that paracetamol could be used effectively in doses of up to 2600 mg/day for two years without significant adverse outcomes [8]. However, there has been a controversy about the gastrointestinal safety of paracetamol, particularly as compared with NSAIDs because of few reports suggesting possible GI side effects from paracetamol [9]. Nevertheless, these data have not been replicated and a recent meta-analysis of RCTs, which avoids channelling bias, showed no more GI symptoms from paracetamol than from placebo [4]. Altogether, the clinical evidence supports the better overall gastrointestinal safety profile of paracetamol compared with nonselective NSAIDs. 

Current European evidence-based recommendations for the management of knee, hip, and hand OA devised by the European League against Rheumatism (EULAR) state that “because of its efficacy and safety paracetamol (up to 4 g/day) is the oral analgesic of first choice and, if successful, is the preferred long term oral analgesic” [2–4]. This recommendation is based on evidence (level 1B) that paracetamol is effective in the treatment of knee OA and that in many patients it is comparable with ibuprofen in the short term and almost as efficacious as naproxen. There is also evidence (level 1B) that paracetamol can be taken safely over the long term. Thus, in agreement paracetamol is available over the counter and frequently used as self-medication for the treatment of mild to moderate pain. 

## 3. Placebo Effects in Osteoarthritis

Placebo is a “sham drug” containing only starch or other inert fillers without any pharmacologically active substance, which is externally indistinguishable from a true drug. As ineffective substances, placebo is primarily used nowadays within the double-blind RCT setting. The placebo itself cannot trigger any pharmacological effect, and therefore their role is to allow a control group to be treated without therapeutic effect. However, other subjective and psychological effects are possible. The term “placebo effect” means this effect of the administration of a placebo.

The placebo effect has long been a source of speculation. A common conception was that effects of placebo interventions are large such as documented by numerous randomized trials in a wide range of clinical conditions. However, this prevailing opinion was questioned when Hróbjartsson and Gøtzsche reported a meta-analysis of 114 randomized trials, across 40 clinical conditions [10]. The challenging results indicated a significant, but modest, effect of placebo on continuous subjective outcomes. Pain was the only condition for which a statistically significant effect of placebo could be observed. An updated analysis including a new sample of trials reproduced the key result of a statistically significant placebo effect for trials with patient-reported continuous outcomes, especially for pain [11]. Further evidence from placebo analgesia experiments strongly supports the reality of the placebo effect in pain trials [12]. Vase et al. [12] compared the clinical trials of pain treatment included in the meta-analysis of Hróbjartsson and Gøtzsche [10] with experimental studies evaluating placebo analgesia that included a no-treatment condition. In the clinical trials, patients were told that they would receive either active treatment or a disguised placebo, whereas in the studies of placebo analgesia patients receiving placebo were told that they would receive a powerful painkiller. Consequently, the effect of placebo analgesia is markedly greater when patients are told that a placebo treatment is a powerful painkiller than when they are told that they may receive either a powerful painkiller or placebo and obviously depending on the informational context and different verbal instructions about certain and uncertain expectations of analgesia [13]. Altogether, the observed placebo effect in RCT has real and apparent components [14]. True effects of placebo administration are unconscious conditioning, conscious expectations, mode of administration and context of administration, the physician's personality, and the doctor-patient relationship. However, simulated effects such as the natural course of the underlying disease and the statistical phenomenon of regression to the mean can mimic the true placebo effects.

Triggered by the discussion about the placebo effect in general and because of the lack of a systematic review of placebo effect in RCTs, Zhang and colleagues recently performed a meta-analysis to examine the placebo effect and its potential determinants in the treatment of OA [5]. The study aim was to determine whether placebo has clinical effects in the treatment of OA, by comparing outcomes at baseline and endpoint and by comparing placebo with untreated control. In addition, they examined possible determinants of the placebo effect in OA. For their analysis, Zhang et al. defined the placebo effect as the change from baseline to endpoint in the placebo group, estimated as the effect size (ES). The ES as found in the placebo group was compared with the ES obtained from untreated controls. As the main result came out that placebo is very effective in the treatment of OA, especially for pain (ES 0.51), stiffness (ES 0.43), and self-reported function (ES 0.49) [5]. The ES for pain in the untreated controls was 0.03. The baseline severity, the expected strength of the treatment, the route of delivery, and the sample size appeared to be important determinants of the magnitude of effect [5]. Further factors such as reporting bias (a patient might feel obliged to report an effect when there really is not any: “I will please”), publication bias, and concomitant treatment might play a role as well (as discussed by Bijlsma and Welsing [15]). Especially, rescue medication is such a factor, which might interfere with the true placebo effect. 

## 4. Rescue Medication in Osteoarthritis Trials

Many patients with OA who are recruited for trials are likely to have exacerbations of symptoms (flares) which require concomitant treatment during the study, irrespective of the type of study design used. Such rescue medications sometimes called “escape medications” are medicines, which the European Medicines Agency (EMA) clinical trial guidance covers under the term Non-Investigational Medicinal Products (NIMPs) [16] to be presented in the study protocol under concomitant medication and secondary endpoints. Rescue medication allows patients to continue in the clinical trial when the efficacy of the Investigational Medicinal Product (IMP) is not satisfactory, for example, placebo controlled clinical trials where a standard treatment is available or dose response studies where lower doses might be ineffective [16]. It is possible to provide single blind rescue medication as well as open-label rescue medication. However, rescue medication use influences the evaluation of symptoms and, thus, complicates the interpretation of results. The main problem of using rescue medication is that alleviated and reduced symptoms can bias the difference in outcome between the placebo and the active treatment group. Furthermore, it is difficult to define the optimum dose when rescue medication is used. Thus, it is crucial to instruct the patients to use the rescue medication only if necessary. 

In OA trials usually short-acting analgesics such as paracetamol or sometimes ibuprofen are given to control acute episodes of breakthrough pain in a patient on a pain management regimen. For example, in a trial on retention on treatment with lumiracoxib and celecoxib, in which a maximum dose of 2 g paracetamol was permitted, rescue medication was used by 79.5% to 81.3% of the patients [17]. The recent meta-analysis examining the placebo effect and its potential determinants in the treatment of OA identified only 15 trials out of 193 trials, which did not allow rescue medications [5]. The EMA clinical trial guidance recommends exposing to rescue medications only those patients assigned to placebo or to an ineffective dose of the treatment; this should minimize the possibility of interactions with the test medicine [16]. According US Food and Drug Administration (FDA) guidance, the effects of confounders such as rescue medication and assistive devices should be standardized in the protocol and in the analysis of OA trials [18]. The EMA demand in the guideline on clinical investigation of medicinal products used in the treatment of osteoarthritis that rescue treatment (including physical therapy) should be standardised, monitored, and carefully recorded for each individual patient [19]. The time points of endpoint assessment should be appropriately chosen to avoid confounding effects of the rescue medication. 

Paracetamol is the rescue medication in most placebo-controlled OA trials. Therefore, the question arises if the guidelines and recommendations are followed. A look into recently published OA trials retrieved by PubMed shows a considerable diversity in dosing, recording, and monitoring of paracetamol use as well as an even larger variability in reporting study results (Table 1) [20–29]. The dose permitted varies between 325 mg per tablet with 1-2 tablets to be taken every 4–6 hours up to 4 g daily. Only in two studies, patients were instructed not to take this drug within 24 hours and 48 hours, respectively, of a follow-up visit to allow for accurate measurement of their current pain levels. Rescue medication was in one study the main outcome and in the others a secondary endpoint or not defined as an out-come parameter. The number of tablets used or the averaged dose was shown in only four trials. Two trials presented not at all results. Altogether, the reporting of rescue medication with paracetamol in OA trials is far from fulfilling the recommendations and limits the validation of the impact on the placebo effect. 

Interestingly, neither the two publications [5, 15] dealing with the placebo effect in OA trials nor most recent extensive reviews [30, 31] on the placebo effect in general including pain studies discussed the possible impact of paracetamol as rescue medication on the observed placebo effect. 

## 5. Paracetamol and Placebo Effect: A Missing Link?

Individuals with greater baseline level of OA pain or use of a limited form of treatment would be more likely to utilize rescue therapy. This was the case in the Glucosamine/chondroitin Arthritis Intervention Trial (GAIT), which allowed up to 4000 mg of paracetamol daily as rescue analgesia. Patients with moderate-to-severe pain used 1.9 ± 1.9 to 2.5 ± 2.2 tablets (500 mg) per day at the end of followup compared to 1.4 ± 1.6 to 1.7 ± 1.8 tablets in patients with mild pain [32]. The primary outcome measure was a response to treatment, defined as a 20% decrease in the summed score for the Western Ontario and McMaster Universities (WOMAC) pain subscale. This outcome was reached in 64% of the glucosamine group, 65% of the chondroitin sulphate group, 67% of the combination, 70% of the celecoxib, and 60% of the placebo group. Overall, differences between placebo and the active treatments were relatively small and reached significance only in comparison with celecoxib. The high rates of response to placebo may relate, in part, to patients' biases and expectations. Another important factor might be the enrollment of patients mostly having relatively mild knee pain at baseline as compared with classic OA studies with a flare design (as discussed by Clegg et al. [32]). Additionally, one may speculate that the use of paracetamol rescue medication in the patients with mild symptoms importantly contributed to the high rate of pain release in the placebo group.

Altogether, paracetamol may be a missing link to explain at least partially the high placebo rate in OA trials not seen in other medical conditions [10]. Indeed, Zhang et al. [5] reported in their meta-analysis that only 15 trials analyzed did not allow rescue medications. The effect size was 0.71 without rescue medication compared to 0.51 in all trials (*n* = 193) and 0.03 in trials with untreated controls (*n* = 14), which suggest that rescue medication can mask the efficacy of the active treatment. However, rescue medication use is not assessed sequentially along with other variables in OA trials and mostly limited or no data are available from published trials on the starting dose, final dose, dose over time of paracetamol rescue medication, and the percentage of patients who used rescue medication during the study. Therefore, there is a need to collect comprehensive data on active use of rescue medication from studies in OA and to evaluate further the contribution of paracetamol as one determinant of placebo effect. Like the natural course of disease and regression to the mean paracetamol may be an important additional simulated effect of placebo administration mimicking the true placebo effect. 

## 6. Conclusion and Future Directions

Paracetamol (350 mg up to 4000 mg) is used as rescue medication in nearly all OA trials. Rescue medication use is not assessed sequentially along with other variables in OA trials. Mostly limited or no data are available from published trials on the starting dose, final dose, dose over time of paracetamol rescue medication, and the percentage of patients who used rescue medication during the study. Paracetamol may be a missing link to explain at least partially the large placebo response in OA trials not seen in other medical conditions except pain studies. The use of rescue medication with paracetamol may play an important role in the interpretation of results of OA trials. Therefore, future OA trials will require the meticulous recording of paracetamol medication use in order to assure homogeneity. Electronic systems such as the Medication Event Monitoring System may aid in recording the consumption of paracetamol objectively [33]. Symptoms and the use of paracetamol should be assessed in a consistent manner, as both are interrelated outcome measures. It appears vital to find a consensus on a standard procedure for evaluating the use of paracetamol as rescue medication in order to draw correct conclusions from placebo-controlled OA trials. 

## Figures and Tables

**Table 1:** Paracetamol rescue medication in randomised controlled osteoarthritis trials in selected recent publications.

Trial	Doses allowed (mg)	Primary or secondary endpoint	Results	Remarks
Frestedt et al. [20]	325 mg per tablet with 1-2 tablets to be taken every 4–6 hours as needed for pain	No	No significant differences between the two groups for rescue medication consumption at any single time point or over time	No figures given

Karlsson et al. [21]	≤4000 mg/day could be taken as for unacceptable pain for more than 24 hours	No	Rescue medication usage was significantly better than under placebo	No figures given

Jacquet et al. [22]	Tablets equivalent to 500 mg paracetamol alone or combined with weak opiates (e.g., coproxamol or coparein)	Main outcome measure 500 mg paracetamol equivalent tablets per week (PET/week) measured each month	Single-component Paracetamol (number of users, tablets/week ± SD) Phytalgic 12 (21 ± 14.7) Placebo 15 (15.4 ± 10.4)	Add-on study to the usual symptomatic medication (analgesics and/or NSAIDs)

Krüger et al. [23]	500 mg tablets; intake was permitted if necessary due to pain but not 48 hours before visits	Secondary endpoint, recorded daily by the patient in the diary and checked by the physician at each visit (pill counting)	Low acetaminophen consumption; differences between the groups not significant	No figures given

Puopolo et al. [24]	For breakthrough pain, if needed; no dose reported	Secondary endpoint; use was determined by tablet counts	Patients treated with etoricoxib or ibuprofen used significantly less paracetamol than those receiving placebo for breakthrough pain Paracetamol use in the etoricoxib and ibuprofen groups was similar	Figures not listed in the table of the key secondary results

Reginster et al. [25]	325 mg tablets; use was restricted (it was not permitted during the initial 2 weeks of treatment) and recorded	No	No data shown	

Sawitzke et al. [26]	Up to 4 g daily could be taken, but patients were instructed not to take this drug within 24 h of a follow-up visit to allow for accurate measurement of their current pain levels	No	The use of paracetamol averaged 570 mg daily. The lowest use was in the celecoxib (465 mg) group and the highest use in the placebo group (645 mg)	Rank order of rescue drug use (least to greatest) exactly paralleled to that of the primary efficacy outcome

Schnitzer et al. [27]	500 mg tablets for use in case of increased OA pain, with a maximum accepted dose of 2000 mg/day	Additional efficacy measures	Average daily tablets use in the placebo group 1.77 versus 1.33 to 1.43 in the naproxcinod groups and 1.34 in the naproxen group	

Yang et al. [28]	Maximum of 4 g/day	No	No data shown	Asked to stop analgesics at least 1 week before completing the questionnaires and visiting their treating orthopaedic surgeon

Thorne et al. [29]	325 mg to 650 mg every 4 h to 6 h as required	Secondary end point; compared for each treatment, based on the average daily consumption, and was summarized each week	Significantly greater use during the placebo phase (3.4 ± 3.6 tablets/day) than during the CR tramadol phase (2.4 ± 3.1 tablets/day)	

